# A Novel Energy Efficient Topology Control Scheme Based on a Coverage-Preserving and Sleep Scheduling Model for Sensor Networks

**DOI:** 10.3390/s16101702

**Published:** 2016-10-14

**Authors:** Binbin Shi, Wei Wei, Yihuai Wang, Wanneng Shu

**Affiliations:** 1 School of Electronics, Suzhou Vocational University, Suzhou 215104, China; 2 Department of Computer Science, University of Texas at Dallas, Richardson, TX 75080, USA; wxw162730@utdallas.edu; 3 School of Computer Science, Soochow University, Suzhou 215006, China; binncn@icloud.com; 4 College of Computer Science, South-Central University for Nationalities, Wuhan 430074, China; shuwanneng@whu.edu.cn

**Keywords:** energy efficient topology control techniques, Emerging Sensor Networks, sleep scheduling scheme, coverage-preserving, cloud model

## Abstract

In high-density sensor networks, scheduling some sensor nodes to be in the sleep mode while other sensor nodes remain active for monitoring or forwarding packets is an effective control scheme to conserve energy. In this paper, a Coverage-Preserving Control Scheduling Scheme (CPCSS) based on a cloud model and redundancy degree in sensor networks is proposed. Firstly, the normal cloud model is adopted for calculating the similarity degree between the sensor nodes in terms of their historical data, and then all nodes in each grid of the target area can be classified into several categories. Secondly, the redundancy degree of a node is calculated according to its sensing area being covered by the neighboring sensors. Finally, a centralized approximation algorithm based on the partition of the target area is designed to obtain the approximate minimum set of nodes, which can retain the sufficient coverage of the target region and ensure the connectivity of the network at the same time. The simulation results show that the proposed CPCSS can balance the energy consumption and optimize the coverage performance of the sensor network.

## 1. Introduction

In Emerging Sensor Networks (ESNs), sensor nodes are equipped with several abilities, including information acquisition, wireless communication, data processing and self-organization. Those nodes can work together to complete a large-scale and complex monitoring task [1,2]. Due to the limited energy, reducing energy consumption as much as possible while meeting the requirements for continuous and effective monitoring is a major issue. The high-density random deployment of nodes in ESNs can ensure high network connectivity and coverage [3], but the problem of this manner is that a large number of redundant nodes could come into being, which will enhance communication interference and reduce the network energy efficiency [4]. At present, the main approach to solve the above problems is to use node sleep scheduling methods [5], whereby the nodes that need to work are selected from among the active nodes, and the other nodes are set to be dormant. Accordingly, the active nodes and non-active nodes work alternately through some reasonable scheme, so that the network lifetime can be prolonged.

Topology reconfiguration technology can not only improve the network coverage performance and connectivity, but also may be used to balance the network energy consumption and thus improve the life cycle of the entire system. Among them, one effective method is to reduce the number of active nodes using a node sleep scheduling scheme. Only when the active node fails, some redundant nodes will be awakened. In the early stage, a certain number of nodes are selected randomly according to the nodes’ sensing distances, and the other nodes can be turned to a dormant state. In order to reduce the uncertainty effects of random scheduling, several works have been implemented to operate a node set rotation method. In this way, all nodes are divided into multiple sets, and each set is rotated independently to undertake monitoring tasks. Once the number of nodes in each set is the least and the number of node sets is maximized, this will extend the time interval of nodes in an active state. The main idea is that the redundant nodes should shut down to save energy after a quantitative selection process, and at the same time the initial coverage of the network is guaranteed.

With a controlled scheduling scheme, some nodes take turns to be in a dormant state with low power, which can conserve the node’s energy and improve the network lifespan. The current control scheduling methods can be classified as follows: random scheduling [6] and sleep scheduling [7].

● *Random scheduling methods*. The nodes can autonomously sense and collect the information of the target area on the basis of a variety of factors, for example, location information, the number of neighbor nodes, node residual energy, etc. [8]. Then, according to the built-in algorithm, the sensor node can decide freely whether it should remain in working condition or close its function module into a state of dormancy. In addition, in clustered ESNs, the cluster head can control the working status of its member nodes by means of the hierarchical structure [9]. Generally speaking, in the stochastic scheduling method, all nodes can determine the appropriate status alone from their own actions, no matter what the other nodes do, which can effectively reduce the communication overhead between nodes.
● *Sleep scheduling schemes*. By making a global and comprehensive judgment of the nodes based on the degree of coverage redundancy in the network, the redundant nodes may be closed according to certain rules so as to achieve a balanced energy consumption and prolong the network lifetime [10]. This type of scheduling approach demands that the nodes cooperate with each other to complete the tasks, and massive factors should be taken into account comprehensively to determine which nodes are redundant, for example, the coverage ratio, nodes’ location information, the number of neighbor nodes, the distance between nodes, etc. [11,12]. In this way, the sharing of state information between nodes will increase communication overhead, but the advantage is that the distribution of active nodes in the target area is more balanced. This can effectively avoid the problem of “routing holes” in some areas of the network which occurs more frequently with stochastic scheduling methods.

By judging whether the node is redundant, the node sleep scheduling scheme can adjust the sleep-wake schedule of eligible nodes and reduce the sensors’ energy consumption [13]. However, it will generate another problem, that is, whether the coverage and connectivity of the network can be guaranteed. ESNs are data-centric networks, and the leading performance indicator of the quality of the network is whether can obtain all the information in the monitored area [14], which is the most essential requirement for the design of ESNs. For example, in event monitoring applications, the primary concern is whether the deployed network can detect all the events occurring within the entire region [15,16]. Besides, the coverage problem is used to describe the non-destructive requirements of the information about the monitoring region. Therefore, an appropriate sleep scheduling method should not be implemented at the cost of affecting the performance of the system. Generally, the sensor nodes with high power may have more possibility to remain in an active state for monitoring and packet forwarding, and some nodes with low power will be put into the sleep state, under the condition of not affecting the network connectivity and providing sufficient coverage of the target area. On the one hand, by reducing the density of active nodes in the network, it can reduce the redundancy of the sensing data and the wireless communication conflicts and interference [17]. Hence, the energy cost of the whole system will decrease. On the other hand, when some nodes fail due to energy depletion, the adjacent sleep nodes can work instead of the failed nodes to maintain the normal operation of the network [18]. Therefore, the node sleep scheduling method must take into account of the following two basic factors: (1) ensure the approximately full coverage of the target area; (2) ensure that all the working nodes of the communication network are still connected.

Furthermore, the goal of topology control is to form an optimized network topology which can extend the life cycle of the network on the condition of ensuring the network coverage and connectivity. In addition, it can improve the performance in aspects of time delay, communication interference, load balance, reliability, scalability, etc. The quality of a network topology is difficult to measure directly by the final goal of topology control. Therefore, it is the first choice for the network design and planning to achieve a good topology in the design of topology control mechanism. In general, the relationship between the various factors of the topology design objectives is complex, so this should be considered and coordinated comprehensively in practice according to the specific application of the network.

In particular, due to the fact ESNs are usually applied in harsh environments or a state of emergency, such as military battlefields and disaster relief, there exist some problems about topology control in aspects of dynamic, adaptive, reliability and survivability as follows:

● The model is too idealistic, and there are many uncertain factors in practical application, which cannot meet the dynamic network topology requirements;
● Lack of effective measurement of the dynamic and self-adaptation network topology;
● Lack of an effective fault tolerance mechanism or method for high reliability and strong survivability in terms of topology control.

The rest of this paper is organized as follows: a brief overview of related works is presented in Section 2. Section 3 discusses the system optimization model, and the coverage-preserving control scheduling scheme is proposed in Section 4. The simulation results and performance analysis is presented in Section 5. Finally, Section 6 concludes the paper and discusses some future research directions.

## 2. Related Works

The main challenge in the design of ESNs is to meet the functional requirements of the network, such as reducing time delays and improving the data integrity. Meanwhile, it should deal with the problems of calculation overhead, energy consumption and communication interference under the constrained resource conditions. At present, ESNs usually use various methods to try to optimize each layer in the communication protocol, including: data fusion [19], hierarchical topology [20], minimizing the idle listening time [21], adaptive node wake up schemes [22], lightweight data authentication and encryption [23], and so on. Among them, the deterministic way by deploying sensor nodes is a common optimization strategy, which can ensure the coverage and connectivity of the area to be monitored by planning the nodes’ density and deploying their position accurately [24]. However, there are a lot of applications where the sensor network nodes are randomly deployed or the distribution of nodes is not uniform. 

In order to achieve a uniform node density, high network coverage and stable connectivity, it is often necessary to adjust or control the nodes’ state. In addition to the performance of coverage and connectivity, the nodes’ location and network topology will affect Quality of Service (QoS) properties, for example, energy consumption, time delay and throughput and so on. Obviously, the communication quality and the throughput of the link will weaken significantly as the distance between the nodes increase excessively, besides, it will increase the energy consumption for packets forwarding.

In [25], an optimized broadcast BPS protocol for sensor networks was proposed, which adopts a geometric-based approach to reduce the retransmissions adaptively by maximizing the hop length. In BPS, nodes did not need any neighborhood information and the approximate optimal connected coverage set is solved by a broadcast and time delay scheme. In [26], an Adaptive Coordination Protocol (ACP) was suggested to determine the deployment of backbone nodes over heterogeneous wireless networks. The main idea was to construct the network structure based on the cellular model, and used the node’s position in the hexagon model as the “strategic point”, then to determine the actual location of the sensor nodes.

Tian et al. [27] proved that k-degree coverage preservation of the target area can be achieved while the communication range is twice the sensing range, and then proposed a method to determine whether the node’s sensing coverage area had been covered by other working nodes. By acquiring the location information of the neighbor nodes in the sensing range, the node can calculate the contribution of circle angle from its adjacent nodes. If the circle angle coverage from its neighbor nodes ran up to 2π, the node can be determined to be a redundant node and switched to a dormant state. 

In order to determine whether the node was redundant more accurately, Huang et al. [28] proposed a coverage degree that can be obtained by means of the ratio of the circumference in the nodes’ edges being covered. In [29], a novel energy consumption model was devised and a heuristic distributed greedy algorithm was proposed to solve the problem of multiple target coverage. The proposed algorithm aimed at maximizing the number of targets by establishing a mathematical model and adjusted the sensing radius of the nodes adaptively to save network energy consumption. With the intention of maintain the connectivity of the network, a Communication Weighted Greedy Cover (CWGC) algorithm [30] was introduced to avoid potential coverage holes.

Aiming at resolving the coverage problem for randomly deployed mobile sensor networks, Meng et al. [31] presented two strategies named coverage-priority and connectivity-priority for node deployment based on a ring model of energy consumption. Fu et al. proposed a novel perception model for three-dimensional space and a coverage-enhancing algorithm for a plane monitoring area [32]. The optimal pitch angle of the node was obtained by the computational geometry method, and then the optimal angle of deflection was calculated by the particle swarm optimization algorithm.

In [33], the minimum cost to achieve full coverage for discrete points on the plane area was implemented under a deterministic deployment, which constructs a mathematical model of an integer linear programming problem. In [34], a distributed C2 algorithm was proposed based on mobilized sensors and a clustering structure. The monitoring region was divided into several hexagonal grids by using the nodes’ communication distance, and the connectivity of the network was guaranteed by adjusting the position of the nodes in the neighboring clusters. Considering that the network cost was minimized while the resulting lifetime was at least equal to a given value, Liu et al. [35] placed the minimum number of sensors that can be deployed to cover the given target objects during a limited time interval. Next, they proved that the problem of covering the target objects completely is NP-hard.

Deng et al. proposed a distance-based on linear sleep scheduling algorithm [36], which was suitable for clustering ESNs with dense sensor nodes. The main idea of the proposed algorithm was to make the cluster member nodes far from the cluster heads have higher probability to enter the dormant state, so as to ensure the quality of network services and reduce the energy consumption of the whole network. Besides, each node can dynamically adjust the transmission power to change its own emission radius, and it can improve the energy efficiency of network nodes, especially for the member nodes close to the boundaries of the cluster. The problem was that the proposed algorithm only applied to static clusters, that is, once the cluster head was selected the structure of the cluster will not be changed.

Wang et al. [37] proposed an integrated coverage and connectivity configuration for energy conservation in sensor networks, which can ensure that the network achieve *K*-coverage and *K*-connectivity while maximizing the number of dormant nodes. The proposed scheme was applied to the convex region, and fully considered the impact of the nodes dispersed around the boundary of the network. In [38], a heuristic algorithm was proposed to select exclusive sets of sensor nodes to cover the monitoring area completely. In addition, the exclusive sets can be updated after a round robin to balance the energy consumption of all nodes. Next, the target coverage problem was regarded as a Maximal Set Cover (MSC) problem and proved the NP-completeness of the problem. Cardei et al. proposed two heuristics based on linear programming and greedy approach to find the maximum number of set covers [39].

Xu et al. [40] introduced a coverage-preserved and connected distributed Voronoi coverage algorithm to detect coverage redundancy sensors and implemented an energy efficient self-scheduling strategy to improve the astringency of distributed scheduling. In [41], an energy-efficient protocol was proposed for deterministic and probabilistic coverage in sensor networks, where the nodes determined their final state autonomously according to a δ-circle and random intervals.

In [42], an optimal deterministic algorithm was proposed based on the target weight by applying the target overlap and greedy algorithm. By using a perceived probability model and the greedy algorithm, the minimum value of indicator function was determined, and the overlap degree of the targets and the optimal deployment location of sensor nodes can be obtained. In [43], an adaptive adjustment of a sensing radius algorithm was proposed to achieve an effective coverage control, in which the nodes chose the optimal coverage adaptively and nodes’ energy consumption could be reduced. Zhao et al. proposed a detect-repair scheme to solve the problem of coverage holes for area coverage in wireless sensor network with a Voronoi algorithm [44]. By using geometry theories and vector algebra to measure the sensing range of nodes, the hole areas can be calculated accurately so as to deploy the least number of nodes to maintain the network connectivity.

## 3. System Optimization Model

### 3.1. One-Dimension Normal Cloud Model

Most of the existing algorithms cannot meet all the requirements such as connectivity, coverage, fewer active nodes and lower computation cost. In this paper, a coverage-preserving control scheduling scheme based on a cloud model and partition of the target area in emerging sensor networks is proposed. By employing the normal cloud model, the degree of similarity between the sensor nodes can be measured in terms of their historical data, and then all nodes in each grid of the target area can be classified into several categories.

For the nodes in the adjacent area, measuring the similarity between them is a very difficult task due to their cognitive behavior and instability. In the similarity analysis of the information monitored by the sensor nodes, most of the algorithms concentrate on the comparison of the data according to some statistical model. However, they do not systematically consider two uncertainties: randomness and fuzziness. Liu et al. [45] introduced the concept of membership cloud and membership cloud generators, which provided a new way of combining fuzziness and randomness. It mainly reflects the fuzziness and randomness of the concept of human knowledge, and provides a new method for the study of artificial intelligence. Also, a cloud model can represent the relationship between randomness and fuzziness, and involve the transformation from qualitative notion to its quantitative instances. Due to the characteristics of randomness and fuzziness, it has been widely used and has achieved good results in various aspects of data mining, knowledge discovery, signal handling and decision analysis.

The normal cloud model can demonstrate the uncertainty between the qualitative model and quantitative model by using linguistic values, which primarily describe the two kinds of uncertainty in the objective world: fuzziness and randomness. Since many phenomena in the real world are subject to or similar to a normal distribution, the normal cloud model is a significant and universal model which possesses good mathematical properties and general applicability. In the similarity analysis of the information monitored by the sensor nodes, most of the algorithms concentrate on the comparison of the data according to statistical model. Then, the prediction of the uncertainty of the model is seldom taken into account. Actually, in the data collection process, a general regularity exists in that real environmental data tends to vary near the trend and close to the normal distribution. This coincides with the classical statistical theory. In this section, we will present the similarity evaluation method based on a normal cloud model to analyze the historical data collected by the monitoring nodes for sensor classification.

**Definition** **1:** Assume U is the universe of discourse, and T is a qualitative concept in U. If x (x ∈ X) is a random instantiation of concept T, which meets μ_T_(x) ∈ [0.1] and has a stable trend. Thus, the distribution of x in the universe T can be defined as a one-dimensional normal cloud.

**Figure 1:**
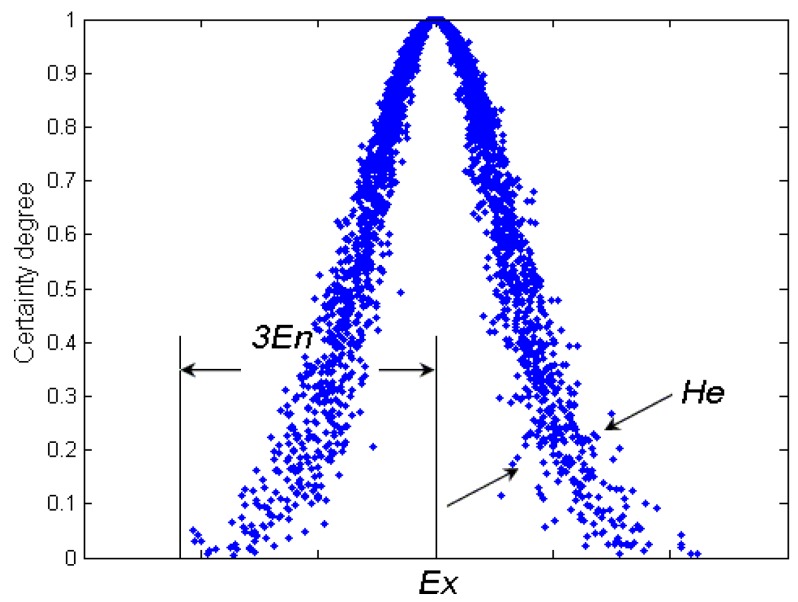
A typical normal cloud model.

The cloud is composed of a plurality of cloud drops, which can be produced repeatedly and are integrated to reflect the overall characteristics of qualitative concept. As is can be seen from Figure 1, the digital features of cloud can be repressed as: expectation parameter *Ex*, entropy parameter *En*, and hyper entropy parameter *He*. The explanation of the above parameters in normal cloud is as follows:

● *Ex* is the mathematical expectation of the cloud, which is the most typical sample to represent a qualitative concept.
● *En* represents the uncertainty metric of a qualitative notion, which demonstrates the level of random dispersion of cloud drops in views of the qualitative concept. It is relevant for both the randomness and the fuzziness of the concept. Mathematically, *En* contributes to represent the average scope of the universe. Besides, with another parameter *He*, they can represent the extent of random dispersion of cloud drops.
● *He* is the uncertainty degree of entropy *En*, which reflects the randomness and fuzziness of entropy and is often used for measuring the uncertainty of entropy and determined by randomness and fuzziness of entropy.

The overall features of the qualitative concepts can be denoted as cloud feature vector *C*(*Ex,En,He*).

The normal cloud is a kind of uncertain transformation model from a qualitative concept to a quantitative representation. Correspondingly, the positive normal cloud generator establishes the mapping relation between the qualitative concept and the quantitative data. From Figure 1, we can see that the normal cloud has obvious geometric features, which can be used to discuss the properties of the normal cloud. The overall characteristics of the cloud can be depicted by regression curves and principal curves, which is from the expectation along the vertical direction and the orthogonal direction, respectively. Since obtaining the analytical formula is complicated it can only be obtained by a linear approximation method.

The target area is divided into a number of square grids, and then the historical data monitored by nodes in each grid can be collected to construct the cloud model. In order to measure the similarity between the nodes dispersed in adjacent fields, the similarity degree of the expected curve is introduced. Assuming the sensor network is located in the square area with the length *M* of the sides, the region is divided into *m* × *m* grids where the nodes are analyzed individually. Suppose *P* = {*p*_1_,*p*_2_,…,*p_n_*} is the data set collected at *n* time intervals and *T* is a qualitative concept of *P*, and there exists a random value of *η* ∈ *μ_T_*(*p_i_*), *η* ∈ [0,1]. In other words, *η* is the distribution of data interval of *p_i_* to membership grade *T*.

To analyze the random cloud drop *x*, normal random number *En*’ with expected value *En* and variance of *He*^2^ is generated by the digital features of the cloud. The probability density function *f_En’_*(*x*) can be expressed as:

(1) $$f_{En^{\prime }}(x)=\frac{1}{\sqrt{2\pi }He}\text{exp}\{-\frac{{\left(x-En\right)}^{2}}{2He^{2}}\}$$

Normal cloud *X* is subject to normal distribution with the expected value *Ex* and variance of *En*’^2^, then the probability density of *X* can be given:

(2) $$f_{X}(x)=\frac{1}{\sqrt{2\pi }\left|En^{\prime }\right|}\text{exp}\{-\frac{{\left(x-En\right)}^{2}}{2H{e^{\prime }}^{2}}\}$$

Next, we can get:

(3) $$f_{X}(x)=\int_{-\infty }^{+\infty }\frac{1}{\sqrt{2\pi }He\left|y\right|}\text{exp}\{-\frac{{\left(x-Ex\right)}^{2}}{2y^{2}}-\frac{{\left(y-En\right)}^{2}}{2He^{2}}\}dy$$

Consequently, the function is a density function of normal distribution *N*(*Ex*^2^,*En*^2^), *He* = 0.

Next, the degree of certainty *Y_i_* can be regarded as a sample of random variable $Y_{i}=e^{-\frac{{\left(x_{i}-Ex\right)}^{2}}{2{\left(En_{i}^{\prime }\right)}^{2}}}$, and the probability density of *Y_i_* can be given as:

(4) $$f_{Y_{i}}(y)=\frac{d(g_{Y_{i}}(y))}{dy}=\frac{1}{\sqrt{-\pi \text{ln}\;y}}$$

where $g_{Y_{i}}=P\left\{Y\leq y\right\}=P\left\{e^{-\frac{{\left(x_{i}-Ex\right)}^{2}}{2{\left(En_{i}^{\prime }\right)}^{2}}}\leq y\right\}$.

If *x_i_* corresponds to the *N* dimension domain space, (*x_i_*,*y_i_*) is a point of *N* + 1 dimension domain space and the set of all points that form cloud (*X*,*Y*). While *x_i_* is one-dimensional, the normal cloud (*X*,*Y*) can be a two-dimensional random variable with the joint probability density:

(5) $$\begin{array}{cc} f_{X,Y}(x,y) & =f_{Y}(y)f_{X}(x|Y=y)\\ & =\left\{ \begin{array}{ll} \frac{1}{2\pi He}\times \frac{1}{\text{ln}\;y}\times \text{exp}\left\{\frac{{\left(x-Ex-\sqrt{-\pi \text{ln}\;y}En\right)}^{2}}{4He^{2}\text{ln}\;y}\right\}, & \left(Ex\leq x<+\infty ,0<y\leq 1\right)\\ \frac{1}{2\pi He}\times \frac{1}{\text{ln}\;y}\times \text{exp}\left\{\frac{{\left(x-Ex+\sqrt{-\pi \text{ln}\;y}En\right)}^{2}}{4He^{2}\text{ln}\;y}\right\}, & \left(-\infty <x<Ex,0<y\leq 1\right) \end{array}\right. \end{array}$$

Since *En*’ ~ *N*(*Ex*,*He*^2^), it can be given that *X* should be a standard normal random variable with mathematical expectation $EX=Ex+\sqrt{-\pi lny}En$. a standard deviation $\sigma =\sqrt{-\pi lny}He$. Therefore, the expected curve of the normal cloud can be obtained as:

(6) $$h(x)=\text{exp}\{-\frac{{\left(x-Ex\right)}^{2}}{\pi En^{2}}\}$$

**Figure 2:**
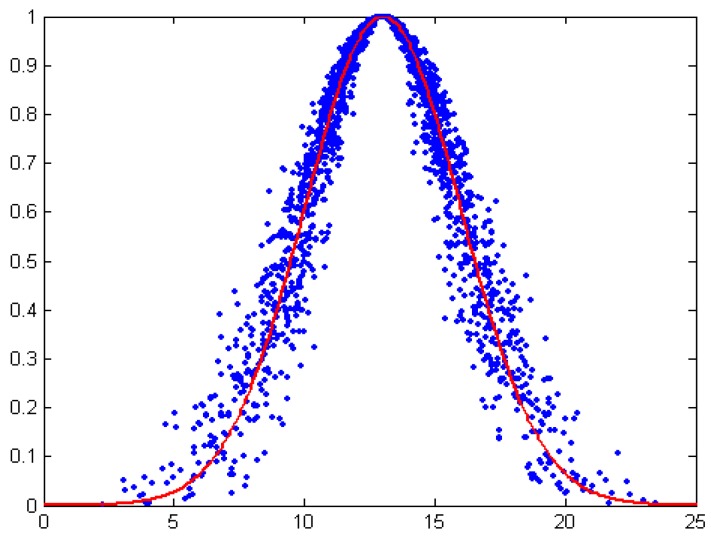
The expected curves of the normal cloud mode.

The degree of dispersion of cloud drops is proportional to *He*, and is inversely proportional to *y*. In other words, *He* is directly proportional to the dispersion of cloud drop, and conversely to the value of *y*. As can be seen from Figure 2, the significant geometric characteristics of the normal cloud can be well reflected by the expected curve method. Besides, all cloud drops fluctuate randomly around the expected curve of normal cloud.

According to Equation (6) and the expression of the desired curve, the expected curves of the two cloud models are:

(7) $$\left\{ \begin{array}{l} h_{1}(x)=\text{exp}\left\{-\frac{{\left(x-Ex\right)}^{2}}{\pi E{n_{1}}^{2}}\right\}\\ h_{2}(x)=\text{exp}\left\{-\frac{{\left(x-Ex\right)}^{2}}{\pi E{n_{2}}^{2}}\right\} \end{array}\right.$$

**Figure 3:**
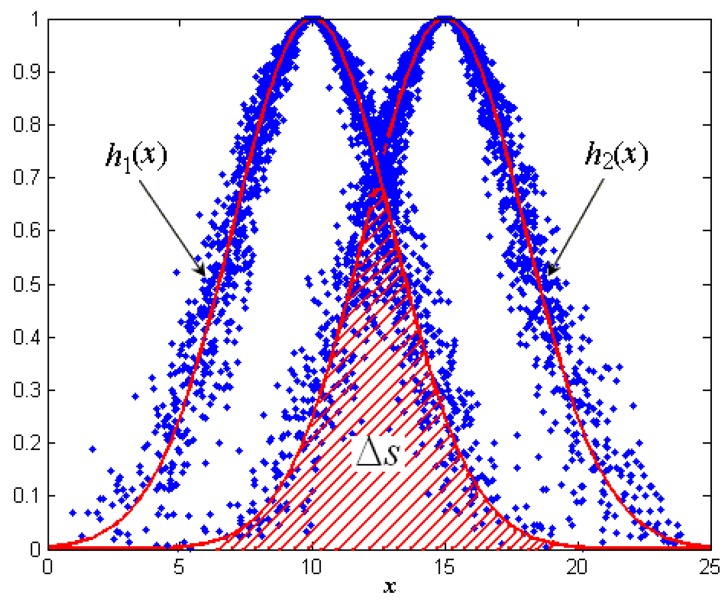
The similarity of two normal cloud models.

Typically, under the premise that the two expected curves are definitive, the area of the overlapping region can be calculated by an integral method. As shown in Figure 3, if the horizontal coordinate of the intersection point of the two desired curve is *x*_0_, then:

(8) $$\Delta s=\int_{-\infty }^{x_{0}}h_{2}(x)dx+\int_{x_{0}}^{+\infty }h_{1}(x)dx$$

where *h*_1_(*x*) and *h*_2_(*x*) are the expectation curve equation for the normal cloud models *C*_1_ and *C*_2_, respectively. This function is a non-integral function, so it can only be approximated by a numerical approximation method. However, the approximate solution method is quite time consuming, which is not feasible to the similarity comparison between plenty of cloud models.

From the expected curve, it is intrinsic that the expression may be approximated by a probability density function of the normal distribution. Then, the expression can be transformed as follows:

(9) $$h(x)=\sqrt{2\pi }En\times f(x)$$

where *f*(*x*) is a probability density function of normal distribution. Next, the relative properties of the normal distribution are used to solve the overlapping area of the two expected curves. Thus, we have:

(10) $$\Delta s=\sqrt{2\pi }\int_{-\infty }^{x_{0}}En_{2}f(x)dx+\sqrt{2\pi }\int_{x_{0}}^{+\infty }En_{1}f(x)dx$$

Suppose θ1=(x0−Ex1)En1 and θ2=(x0−Ex2)En2, then Equation (10) can be expressed as:

(11) $$\Delta s=\sqrt{2\pi }En_{2}\int_{-\infty }^{\theta_{2}}\phi (x)dx+\sqrt{2\pi }En_{1}\int_{-\infty }^{\theta_{1}}\phi (x)dx$$

Compared to the conventional model, the cloud model involves the transformation of a qualitative notion and its quantitative instances, and can systematically consider the randomness and fuzziness of eutrophication assessment. The normal cloud model is the most rudimentary model for its adoption of normal distribution and membership function, which should be satisfied by the node characteristics of wireless sensor networks.

### 3.2. Similarity Criterion and Node’s Classification

In this section, we will discuss how to obtain the redundancy degree of a node with respect to its sensing area being covered by neighboring sensors. Then, a centralized approximation algorithm based on the partition of the target area is designed to obtain the approximate minimum set of nodes, which can retain the sufficient coverage of the target region and ensure the connectivity of the network.

Many methods have been put forward to prolong the lifetime of networks. The most common techniques include: data fusion coding [46], balancing network energy consumption by routing protocol [47], eliminating the redundant data [48], etc. In practical applications, especially in environmental monitoring, a large number of sensor nodes is usually deployed randomly in the target area for ensuring that the monitoring region has sufficient coverage. However, the problem of node redundancy may be impractical to take into account and a lot of redundant nodes will be generated. The approach of searching for these redundant nodes and making them sleep intermittently may reduce the redundant data in network and channel congestion. Further, an appropriate scheduling mechanism can balance the energy consumption of the sensor nodes, and improve the energy efficiency.

The fuzzy clustering technique adopts a fuzzy matrix to classify objects, and it is particularly effective when the boundaries between clusters of data are ambiguous. Different classification results can be obtained for different levels of confidence, and this forms a dynamic clustering chart. For all nodes within a single grid, the normal cloud model is established according to the historical data. By using the correlation property of the normal distribution, the overlapping area of any two nodes can be solved. Firstly, the matrix *SS* = (*s_ij_*)_*n*×*n*_ can be constructed as:

(12) $$SS=\left[\begin{array}{llll} s_{11} & s_{12} & \cdots & s_{1n}\\ s_{21} & s_{22} & \cdots & s_{2n}\\ \cdots & \cdots & \cdots & \cdots \\ s_{n1} & s_{n2} & \cdots & s_{nn} \end{array}\right]$$

Moreover, in order to make the original data mathematically tractable for fuzzy clustering, the original data matrix is needed to be standardized and transformed into fuzzy matrix via proper data transformation. The normalization formula is given by:

(13) $$r_{ij}=\left\{ \begin{array}{ll} 1, & \left(i=j\right)\\ \text{exp}\{-\sum_{k=1}^{n}\left|s_{ik}-s_{jk}\right|\}, & \left(i\neq j\right) \end{array}\right.$$

Therefore, the fuzzy similar matrix can be obtained:

(14) $$R=\left[\begin{array}{llll} 1 & r_{12} & \cdots & r_{1n}\\ r_{21} & 1 & \cdots & r_{2n}\\ \cdots & \cdots & \cdots & \cdots \\ r_{n1} & r_{n2} & \cdots & 1 \end{array}\right]$$

According to the characteristics of the fuzzy similar matrix, there must be a minimum number of natural *k* (*k* ≤ *n*) which the condition of transitive closure satisfies *t*(*R*) = *R^k^* and *R^l^* = *R^k^* for all natural numbers *l* greater than *k*.

Hence, the transitive closure of *R* can be calculated by the square method. Then, it is needed to find the *λ*-truncation matrix of the fuzzy similar matrix, and then the classification of *U* can be given at the level *λ*. In the fuzzy clustering analysis, different classification can be obtained for different *λ* ∈ [0,1]. Here, taking into account the characteristics of similarity of the data collected by adjacent sensor nodes of ESNs, *F*-statistics is employed to determine the optimal values.

According to the original matrix *SS*, let $\bar{s}=\left\{{\bar{s}}_{1},\ldots ,{\bar{s}}_{i},\ldots ,{\bar{s}}_{n}\right\}$ denote the center vector of the population sample, ${\bar{s}}_{i}=\sum_{j=1}^{n}s_{ji}/n$. Suppose that *n* sensor nodes in the grid are classified into different categories according to the value of *λ*, the number of categories is *t* and *n_j_* denotes the number of nodes in the *j*-th category, $\sum_{j=1}^{t}n_{j}=n$. The sample of category *j* is denoted by ${s_{1}}^{\left(j\right)},{s_{2}}^{\left(j\right)},\ldots ,{s_{n_{j}}}^{\left(j\right)}$, then the center clustering vector of the *j*-th category is ${\bar{s}}^{\left(j\right)}=\left({{\bar{s}}_{1}}^{\left(j\right)},{{\bar{s}}_{2}}^{\left(j\right)},\ldots ,{{\bar{s}}_{n}}^{\left(j\right)}\right)$, s¯u(j)=∑v=1njsuv(j)nj,u=1,2,…,m.

Hence, the *F*-statistic can be obtained:

(15) $$F=\frac{\sum_{j=1}^{t}n_{j}{\left\|{\bar{s}}^{\left(j\right)}-\bar{s}\right\|}^{2}/(t-1)}{\sum_{j=1}^{t}\sum_{u=1}^{n_{j}}{\left\|{\bar{s}}_{u}^{\left(j\right)}-{\bar{s}}^{\left(j\right)}\right\|}^{2}/(n-t)}$$

After clustering, the nodes in a single grid are divided into several categories, and the nodes with similar average observation results are classified into a same class. Intuitively, the less nodes determined as redundant nodes and then put into sleep mode, the greater the amount of information retained. For the selection of redundant nodes, we will comprehensively take into account multiple factors, such as the category of the node, residual energy, redundancy degree. In the next section, a highly distributed and scalable mechanism is proposed to increase network lifetime as well as maintain sufficient sensing coverage and the complete connectivity of the network.

### 3.3. Redundancy Degree of Nodes

In most ESN applications, the coverage rate is a key to demonstrate the capacity of monitoring the environment [49], and it also serves as a QoS standard for evaluating the effectiveness of data collection. Actually, small sensing holes do not affect the performance of the entire network. Therefore, partial coverage redundancy is used to measure the redundancy of nodes, which is more suitable for ESNs [50].

To judge whether the node may enter into the dormant state, it is necessary to determine the redundancy degree of the node. In some algorithms, we must know the geographic information of the node and the relative position of its neighbor nodes to calculate the degree of redundancy. Unlike these deterministic methods, the mathematical model was based on the theory of probability [51], and the expected value of a node’s redundancy can be calculated in accordance with the number of valid neighbors and the degree of its neighbors’ coverage.

We consider a wireless sensor network of *n* arbitrarily distributed sensor nodes, whose location information is unknown. All nodes will no longer move after deployment. In addition, the node’s perception model adopts the Boolean model based on circular region, where the coverage area of each node can be confined to a circle about its position with a sensing range *r*. The sensing range of all nodes can be adjusted adaptively, and the sensor-to-sensor distance is obtained by using the signal strength indicator or time of arrival methods. Let *R_i_* and *C_i_* be the sensing range and communication range of node *N_i_,* respectively, and the relationship between them is defined as *C_i_* = *λR*, 1 ≤ *λ* ≤ 2.

**Definition** **2:** The sufficient condition for the communication between the nodes is that the distance is less than or equal to the minimum communication radius, i.e., d(i,j) ≤ min{C_i_,C_j_}.

**Definition** **3:** *Only when the distance between the nodes N_i_ and N_j_ satisfy d(i,j)* ≤ *R_i_ + R_j_, those nodes are neighbors and can communicate with each other directly.*

**Definition** **4:** If each point in a region of a node is covered by at least one of its neighbors, then this area can be regarded as the area of redundant coverage. The redundancy of a node is defined as the ratio of the redundant coverage area by its neighbors to the sensing area of the node.

**Corollary** **1:** For any node i, there exists the neighbor set with the sensing range R_i_. Assuming an arbitrary point x in the sensing area, if the probability of being covered by a neighbor node is denoted by Pr(x), there must be Pr(x) of sensing area covered by neighbor nodes of i.

**Proof:**  Assuming there are *K* perception points {*x*_1_,*x*_2_,…,*x_K_*} in the sensor field of radius *R_i_* for node *N_i_*, the perception points’ distributions are uniformed and the events that the points being covered by neighbor nodes is independent. Considering the probability that a sensing point is covered by its neighbor node is described by *Pr*(*x*), then, the possibility that there are *k* out of *K* nodes being covered by neighbor nodes based on the probabilistic model is given by:

(16) $$P\{X=k\}=C_{K}^{k}Pr{\left(x\right)}^{k}{\left(1-Pr(x)\right)}^{K-k}$$

□

From Equation (16), it should be pointed out that the event is subject to binomial distribution, namely *X* ~ *B*(*Pr*(*x*)*K*). By solving for the mathematical expression of *X*, we have:

(17) $$E[X]=\sum_{k=0}^{K}nC_{K}^{k}Pr{\left(x\right)}^{k}{\left(1-Pr(x)\right)}^{K-k}=KPr(x)$$

So, in the sensing range of *R_i_*, there are about *KPr*(*x*) perception points covered by neighbor nodes. When *K* tends to infinity, the sensing area with the radius $\pi {R_{i}}^{2}$ can be seen as an infinite number of sensing points. Therefore, the probability of the perception points being covered by the neighbor nodes can be regarded as the ratio of the total covered area, i.e.,

(18) $$\underset{K\to \infty }{\text{lim}}\frac{KPr(x)}{K}=Pr(x)$$

Let *Sx* = {*X*_1_,*X*_2_,…,X*i*,…} be the set of perceptual points in the sensing field of node *N_i_* and *x* be the distance from the point to the node. In terms of the Corollary 1, if the sensing set can be covered by its neighbor nodes, the node *N_i_* can be covered by its neighbor nodes completely. Assuming that *Y* is a random variable used to represent the Euclidean distance between the node and a perceptual point *x* within its range, the probability density function of *x* can be calculated as:

(19) $$f_{Y}(x)=2x/{\lambda_{i}}^{2}R_{i}^{2}$$

**Figure 4:**
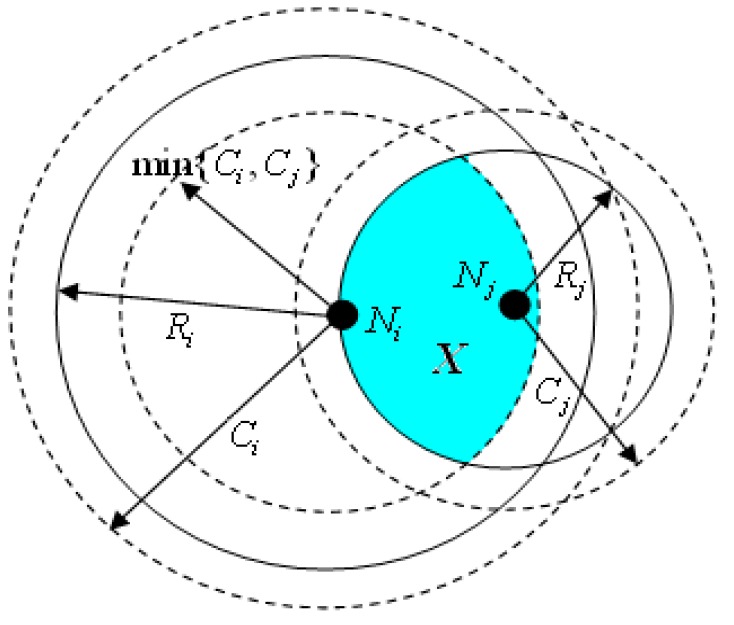
The point within the sensing range of *N_i_* being covered by its neighbor.

As shown in Figure 4, the sensing area *ρ*(*N_i_*,*R_i_*) is the range of the node’s perception. For the point *X* within the range of node *N_i_*, the probability that the point is covered by the neighbor node *N_j_* is denoted *Pr_i,j_*(*x*). If *X* ∈ *ρ*(*N_i_*,*R_i_*) and the point is covered by another neighbor’s node *N_j_*, we can determine that the distance from point *X* to node *N_j_* is less than the sensing range of *N_j_* , meanwhile, these two nodes are able to communicate with each other. Thus, *Pr_i,j_*(*x*) is equal to the ratio of the shaded part within the sensing range *R_j_* to the communication range of node *N_j_*.

The probability *Pr_i,j_*(*x*) of perception point *X* within the sensing range of *N_i_* being covered by the neighbor node *N_j_* can be expressed as follows:

(20) $$\begin{array}{l} Pr_{i,j}(x)=\frac{\rho (X,R_{j})\cap \rho (N_{i},C_{i})\cap \rho (N_{j},C_{j})}{\rho (N_{i},C_{i})\cap \rho (N_{j},C_{j})}\times \frac{f_{Y}(x,N_{i})}{f_{Y}(x,N_{j})}\\ =\frac{\rho (X,R_{j})\cap \rho (i,\text{min}\{C_{i},C_{j}\})}{\rho (N_{i},\text{min}\{C_{i},C_{j}\})}\times \frac{{\lambda_{j}}^{2}R_{j}^{2}}{{\lambda_{i}}^{2}R_{i}^{2}} \end{array}$$

**Figure 5:**
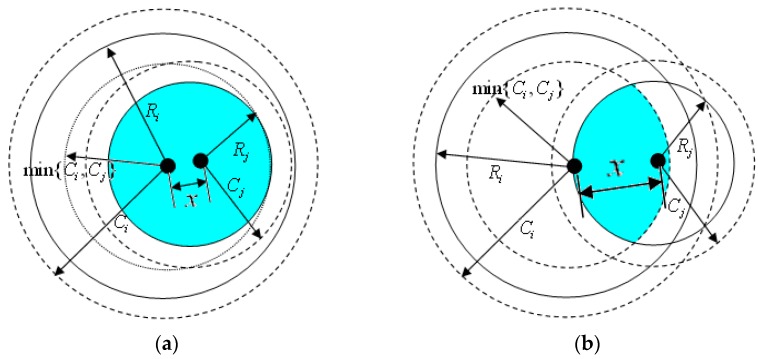
The maximum distance between point *X* and the neighbor node. (**a**) *x* < min{*C_i_*,*C_j_*}; (**b**) min{*C_i_*,*C_j_*} − *R_j_* ≤ *x* ≤ *R_j_*.

According to the previous definition, the sensing range of a sensor node is less than the communication range. Then, we can discuss the problem in two scenarios as shown in Figure 5a,b, respectively.

If 0 < *x* < min{*C_i_*,*C_j_*} − *R_j_*, the maximum distance between point *X* and *N_j_* is about min{*C_i_*,*C_j_*}. But if min{*C_i_*,*C_j_*} − *R_j_* ≤ *x* ≤ *R_j_*, the distance between them can be obtained as [min{*C_i_*,*C_j_*} − *R_j_*,*R_i_*]. Therefore, *Pr_i,j_*(*x*) can be expressed as:

(21) $$Pr_{i,j}(x)=\left\{ \begin{array}{l} \frac{\rho (X,R_{j})}{\rho (i,\text{min}\{C_{i},C_{j}\})}\times \frac{{\lambda_{j}}^{2}R_{j}^{2}}{{\lambda_{i}}^{2}R_{i}^{2}},0<x<\text{min}\{C_{i},C_{j}\}-R_{j}\\ \frac{\rho (X,R_{j})\cap \rho (s_{i},\text{min}\{C_{i},C_{j}\})}{\rho (i,\text{min}\{C_{i},C_{j}\})}\times \frac{{\lambda_{j}}^{2}R_{j}^{2}}{{\lambda_{i}}^{2}R_{i}^{2}},\text{min}\{C_{i},C_{j}\}-R_{j}\leq x\leq R_{j} \end{array}\right.$$

Suppose the point *X* can be covered at least one node with the same category of *N_j_*, then the probability is:

(22) $$\tilde{P}r_{i,j}(x)=1-{\left(1-{\text{Pr}}_{i,j}(x)\right)}^{n_{j}(i)}$$

where *n_j_*(*i*) is the number of sensor nodes with the same category of *N_j_*.

Let *ψ*(*X*) be the random event that point *X* within the scope of *N_i_* being covered by at least one sensor node of a different category. Then, for total *t* categories of node sets, the probability of point *X* being covered by a neighbor node with other categories in the range of *N_i_* can be given as:

(23) $$\tilde{P}r_{i}(\psi (X))=1-\prod_{1\leq k\leq t}{\left(1-{\text{Pr}}_{i,k}(x)\right)}^{n_{k}(i)}$$

Thus, when *N_i_* is covered by at least one neighbor node, its redundancy degree can be calculated by:

(24) $$\iint_{\rho (i,R_{i})}\tilde{P}r_{i}(\psi (X)))d\rho$$

According to the definition of node redundancy, the expected value of the redundancy of the node *N_i_* is the ratio of the area covered by other nodes to its sensing area. Therefore, the expected value of the redundancy is:

(25) $$\begin{array}{cc} E[\phi_{i}] & =\frac{1}{\rho (N_{i},R_{i})}\iint_{\rho (i,R_{i})}\tilde{P}r_{i}(\psi (X)))d\rho \\ & =\frac{2\pi }{\pi R_{i}^{2}}\int_{0}^{R_{i}}\tilde{P}r_{i}(\psi (X)))xdx \end{array}$$

Therefore, the node’s redundancy degree can be obtained as:

(26) $$\begin{array}{cc} E[\phi_{i}] & =\frac{2}{R_{i}^{2}}\int_{0}^{R_{i}}(1-\prod_{1\leq k\leq t}{\left(1-Pr_{i,k}(x)\right)}^{n_{k}(i)})xdx\\ & =1-\frac{2}{R_{i}^{2}}\int_{0}^{R_{i}}(\prod_{1\leq k\leq t}{\left(1-Pr_{i,k}(x)\right)}^{n_{k}(i)})xdx \end{array}$$

## 4. Coverage-Preserving Control Scheduling Scheme

### 4.1. Determination of Redundant Nodes

How to prolong the network lifetime as long as possible under the condition of limited sensor node energy is the main challenge in designing ESNs. The reduction of the number of working sensors is an important and effective means to achieve the above purpose. That is, without impacting the performance of the system, some nodes are scheduled into sleep status to retain their residual energy, and others remain in an active state. In this way, we can reduce the node density of the network, lower the redundancy of monitored information, and cut down interference levels. Thus, the energy consumption of the whole system can be improved. In addition, when a node fails due to energy depletion, its neighboring nodes in sleep status can be activated to replace the failed node, and thus continue to maintain the network coverage. Therefore, density control can effectively prolong the lifetime of wireless sensor networks. Aiming to maintain the original performance of the network, density control must meet the following two conditions: (1) ensure coverage quality of the target area; (2) ensure the connectivity of the network.

**Definition** **5:** Given a set of sensor nodes IS, G_s_ = (V_s_,E_s_) is an undirected graph composed of all nodes in the set, in which V_s_ = S, ∀N_i_,N_j_ ∈ S and dis(i,j) ∈ E_s_. If and only if dis(i,j) ≤ min{C_i_,C_j_}, it can be denoted that the transmission graph G_s_ is constructed by all nodes from set S. Moreover, a transmission path Path = {N_1_,N_2_,N_3_,…} derived from G_s_ consists of a series of nodes, in which every pair of adjacent nodes are communication neighbors. If there exists at least a communication path between any two nodes in the transmission graph G_s_, it is said that the graph is connected.

**Definition** **6:** Given a sensor node set RS and target area R, If every point within the R is covered by at least one of the nodes within the set S, the S is denoted as the cover set of R. Next, meanwhile if the transmission graph derived from the set S is connected, the set S can be denoted as a connected cover set of R.

Considering that a sensor network deployed in the target area *R*, the problem of the minimum connected cover set is to find out the minimum subset S* (S* ⊆ *S*), which $R\subseteq \cup_{N_{i}\in S^{*}}\rho \left(N_{i},R_{i}\right)$ and the transmission graph derived from S* is connected completely. In [52], the minimum connected cover set problem was proved to be a NP-hard problem.

Since that the number of redundant nodes that can be hibernated at the same time determines the size of the final set, we can construct the minimal connected cover set according to multiple criteria, including node redundancy degree, the residual energy, node category, etc. In this paper, a greedy algorithm is used to find the redundant nodes set which can be scheduled to sleep simultaneously. Firstly, a node with the maximum redundancy degree in each grid should be selected and put into the temporary node set. Secondly, the temporary redundant nodes will be closed and at least one node of each type of its neighbors must be in a working state after being selected to the largest independent set. In order to make the final coverage set as small as possible, the above process iterates until no redundant nodes can be closed. At the end of the algorithm, all the redundant nodes are selected and form the maximum independent set. The rest of the nodes will remain in working state in the next round and form the coverage of the target area. Algorithm 1 is described as follows:
**Algorithm 1:** Determination of redundant nodes**Input:** initialize the sensor node set *S* = {*N*_1_,*N*_2_,…,*N_i_*,…} and target area *R*;**Require:** redundant node set *RS*, maximum independent set *MIS*, temporary redundant node set *TS*, temporary active node set *AS*.**Output:** minimum connected cover set (*MCS*) of *R*.1. *RS* = *AS* = ∅;2. *TS* = *S*;3. for each node *N_i_* ∈ *IS* do4. calculate the node redundancy degree;5. broadcast the HELLO message to its neighbors, and receive the reply;6. update the neighbor nodes list;7. obtain the categories of the neighbor nodes;8. end for;9. if ($R⊄\cup_{N_{i}\in MCS}\rho \left(N_{i},R_{i}\right)$ then10. return *MCS* = ∅;11. end if;12. while (TS ≠ ∅)13. *MIS* = ∅;14. obtain the maximum redundancy degree {$max_{N_{i}\in TS}\left\{E\left[\phi_{i}\right]\right\}$};15. for each node *N_i_* do16. if $E_{res}\left(N_{i}\right)>\frac{\sum_{N_{j}\in TS}E_{res}\left(N_{j}\right)}{\left|TS\right|}$ then17. *AS* = *AS* ∪ {*N_i_*};18. calculate the maximum independent *MIS*;19. *RS* = *S* − *AS*;20. end if;21. end for;22. for each node *N_j_* ∈ *RS* do23. estimate the total cover area;24. end for;25. end while;26. return *MCS* = *S* − *AS*;


### 4.2. Control Scheduling Scheme

After the selection of redundant nodes, we need to design a reasonable scheme for scheduling as many nodes as possible to enter the dormant state without creating blind spots. Meanwhile, in order to maximize the network lifetime, the sensor nodes should take turns to enter the dormant state. The scheme has the advantages of less active nodes and uniform distribution, which satisfies the requirements of low sensor node utilization rate and a high coverage ratio.

First, the network is initialized, and each node broadcasts its position information and energy information to its neighbor nodes. The subsequent network lifetime is divided into a number of equal time intervals (Round), and each round includes two phases: scheduling phase and steady phase. Furthermore, the scheduling phase consists of two substages: neighbor discovery and autonomous judgment. In the neighbor discovery phase, the nodes exchange information with neighbor nodes to obtain their location and residual energy, and then establish the neighbor node table. On the basis of the above, the node can decide whether to satisfy the redundant condition. If it does not meet the conditions, the node remains active in the stable stage during the current round and is responsible for target sensing, monitoring and data transmission. Otherwise, the node enters the dormant state until the next scheduling phase. Intuitively, the stable phase is much longer than the schedule phrase, so as to reduce the overhead of the scheduling scheme.

During the neighbor discovery phase, the node firstly broadcasts a HELLO message to its neighbors, which contains the node ID, location information and residual energy. To avoid channel conflict and packet loss while the packets are transmitted simultaneously between several nodes, each node waits for a period of time before broadcasting HELLO messages.

In the node autonomous judgment stage, concurrent dormancy where a number of nodes that are dependent on the cover to enter the state of dormancy at the same time may cause blind spots. In order to avoid the above situation, the scheduling scheme based on priority is introduced. After the neighbor discovery phase, the maximum value of the residual energy of each node denoted by *E_max_* is obtained. In terms of the residual energy and *E_max_*, each node calculates the back off delay:

(27) $$T_{backoff}(i)=\omega \times E_{res}\left(i\right)/E_{\text{max}}+(1-\omega )\times rand(0,1)\times T_{s}$$

where *w* is regulation parameter and *rand*(0,1) represents a random number within the interval [0,1]. Here, the purpose of the introduction of random number is to avoid the same delay being set by the nodes with the same residual energy.

After a period of time delay, each node begins to judge whether or not to sleep. The dormant node broadcasts SLEEP signaling to its neighbor node. Then, the neighbor node will remove dormant nodes from its neighbor nodes table after receiving the corresponding SLEEP signals, and determine the redundant degree again based on the updated neighbor node set. It is obvious that the nodes with lower residual energy will have more opportunities of being scheduled to a sleep state, which can play a role of balancing the energy consumption of the network. After being determined as active or dormant state in next round, the node will remain in the state for a period of time. After *T_w_* time periods, dormant nodes are awakened and all nodes go into the next round. The specific steps are as follows:

*Step 1*: Initialization, all valid nodes are set to Ready state.
*Step 2*: By using the method of Section 4.1, the redundant measurement is adopted to determine whether the redundant node.
*Step 3*: If the current node is a redundant node, it sends a pre-SLEEP message to its neighbor nodes and enter the pre hibernation state. Furthermore, it will start a delay timer *T_backoff_* to listen for more messages. If receiving the pre-SLEEP message from the adjacent nodes in interval *T_backoff_*, return to Step 2. Otherwise, if not receiving the wake-up message in the same interval, the node continues to enter the sleep state.
*Step 4*: After a period of time, the node *i* of the Ready state broadcasts its position information and residual energy to its active neighbors. Then, it will collect neighbor nodes’ information and establish its neighbor node table. According to the residual energy of nodes, the time delay is calculated.
*Step 5*: After a period of *T_backoff_*(*i*), the steady state node begins to estimate the distance between its neighbor nodes, finds the nearest active neighbor nodes and adds it to the neighbor node table.
*Step 6*: Data collection and forwarding.
*Step 7*: Until the time *T_w_* arrives, all dormant nodes will be woken up. Return to Step 2 and prepare for scheduling the next round.

### 4.3. Fault-Tolerant Backbone

The main objective of topology management in wireless sensor network management is to ensure the network’s connectivity and optimize the path for data transmission. Moreover, once failed nodes appears in the network, the practical topology control can adjust the communication links dynamically as far as possible to ensure the normal operation of the network. The topology management can be achieved through the deployment of nodes. For the unattended network with random deployment of nodes, topology management protocols or algorithms should be designed to improve the network performance.

After constructing the maximal connected set of active nodes in each grid, the high coverage and connectivity between nodes can be guaranteed. Next, the virtual backbone should be constructed to forward the data collected in each grid to the sink with respect of ensuring the connectivity of the global network. Thus, it can effectively reduce the energy consumption and prolong the network lifetime. The most conventional technique applied of constructing virtual backbone network is to obtain a Connected Dominating Set (CDS). Most of the data transmission or forwarding occurs between the dominating nodes in multi-hop mode. If there are multiple paths between any source node and the destination, a node can change its choice of next hop once a path failure occurs. In this way, it is necessary to construct a virtual backbone network with fault tolerance. Actually, the centralized algorithms often require global information of the sensor nodes, thus it is hard to be achieved in most cases. In contrast, the distributed algorithms can be extended to the whole network only by local information. In this section, the two-connected dominating set algorithm is proposed to implement the architecture of distributed backbone network, which can ensure the node information collected in different regions is transmitted to the sink node through multi-hop mode.

**Definition** **7:** *Given a graph G =* (*V,E*)*, and suppose a subset of vertexes D ⊆ V. If each vertex in V − D is dominated by at least k directed vertex in D, D is denoted as a distance k-dominating set of graph G. The vertex in D is described as dominator node, and the vertex in V − D is a dominated node correspondingly.*

**Definition** **8:** *Given a graph G =* (*V,E*)*, and suppose a dominating set of vertex C and C ⊆ V. If there exist at least two different paths between each pair of nodes in C, and meanwhile C is a distance k-dominating set of graph G, then C can be denoted as a two-connected k-dominating set of graph.*

The main steps for constructing two-connected *k*-dominating sets for a fault-tolerant backbone is as follows: (1) for network graph *G*, the *k*-dominating set *D* is defined, which can be obtained by a distributed manner based on localized management; (2) The minimum spanning tree of the *k*-dominating set *D* is constructed, and the nodes in the path is added to the set *D*; (3) the nodes of *V* − *D* can be labeled as backbone nodes, which satisfy two-connectivity for all dominated nodes.

During the neighbor discovery phase, each node needs to broadcast HELLO messages to its neighbors for exchanging information. Each node creates a local neighbor list, which includes all one-hop neighboring nodes with respect to their distance. After the exchange of information, each node can obtain the comprehensive information, such as, neighbors ID, estimated distance, color status, etc. Next, the graph coloring algorithm is put to use to color all nodes, which can determine the node’s state being dominating or dominated. Here, black represents the dominating node; gray represents the dominated node and all nodes initiate as white nodes.

Each node contains two parameters of *SUM* and *dom*. For node *u*, the *u.dom* is set to *k* initially. In every round, a node will be selected to the dominating set. If *u* is the direct neighbor of any dominated node, *u.dom* should be updated by subtracting the number of neighbor node in dominating set. Otherwise, *u.dom* can keep constant. Once the node is *k*-dominated or is selected as the dominated node, *u.dom* will be set to zero. *u.SUM* is the sum of the *dom* of all the neighbors around the node *u*. Algorithm 2 is described as follows:
**Algorithm 2:** Constructing two-connected *k*-dominating sets for fault-tolerant backbone**Input:** Graph *G* = (*V,E*), integer *k***Output:** A two-connected *k*-dominating set *C*1. for each node *ν* ∈ *V*(*G*) do2. color *ν* as white;3. *ν.dom* = *k*, *I* = ∅, *W* = *V*(*G*);4. broadcast *HELLO* message and find all neighbor nodes;5. restore the neighbor information (ID, State_color) to the local *neighborLIST*;6. update *pathLIST*;7. end for;8. for each node *ν* ∈ *V*(*G*) do9. for each node *u* ∈ *neighborLIST*(*ν*) do10. if *u.color* = white and *u.SUM* > *MAX_ID* then11. *ν.dom*−−;12. *MAX_ID* = *id*(*u*);13. color *u* as black, and set *I* = *I*∪{*u*}, *W* = *W* − {*u*};14. end if;15. end for;16. end for;17. while W ≠ ∅ do18. *C* = *I* ∩ *W*;19. for each *c* ∈ *C* do20. suppose *c* is *k*-dominated node from set *I*;21. if *dist*(*c*,*MAX_ID*) < *r_c_* && |*neighborLIST*(*c*)| ≥ *k* then22. pop out the vertex *c* from *I*, and set *D* = {*c*};23. else24. *C* = *C* − {*c*};25. end if;26. end for;27. end while;28. for *ν* ∈ *I* and *ν* is not a cut-vertex do29. for *u* ∈ *C* ∪ *W* do30. *P_u,v_* = *shortPATH*(*G*:*u*,*ν*) and *P* = *P* ∪ *P_u,v_*31. put the backbone node on the shortest path to the CDS *D*;32. end for;33. end for;34. return *D*;


## 5. Simulation and Results Analysis

To verify the validity of the proposed scheme, we will compare the performance of CPCSS, Energy Aware Evolutionary Routing (EAER) [53], and Energy Efficient Heterogeneous Clustered (EEHC) [54]. We set up the model and experimental environment, and each examination was run 10 times to obtain the average results so as to eliminate the error caused by randomness. In order to implement senses’ classification, we adopted the data sets from the Intel Berkeley Research Lab [55], where actual sensor nodes are deployed in the lab, during the data sensing process. The data collected by sensor nodes includes the humidity, temperature, light, and voltage values and so on. We randomly choose part of the nodes’ temperature, humidity, and light data, and attach to all the sensors in the experiment, and generate the predicted data sequence for our tests. 

**Figure 6:**
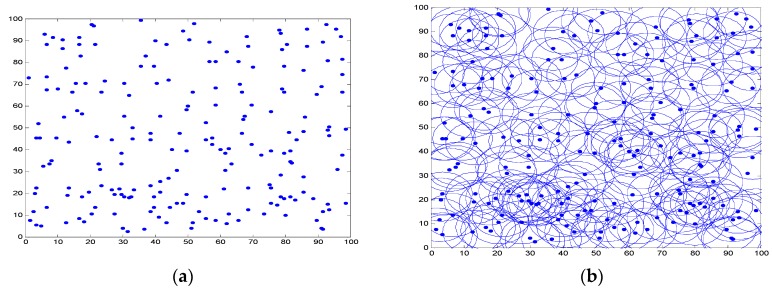
The initial deployment of nodes. (**a**) Nodes’ deployment; (**b**) Coverage area.

There are 200 sensor nodes deployed randomly in a 100 × 100 2D plane, and the node’s sensing range can make adaptive regulation in the range of 9 m to 12 m. Figure 6a,b indicates the active node distribution and active node coverage after the initial deployment of the network, in which the solid points are represented as active nodes in the network. From the nodes’ deployment, it can be observed that most parts of the area are covered by more than one sensor node. Moreover, lots of redundant nodes will lead to produce overload data and mass collision in wireless channel. Intuitively, if those sensor nodes are off-duty when they have no sensing or relaying work to do, the whole system will can achieve a better performance in aspects of energy consumption.

**Figure 7:**
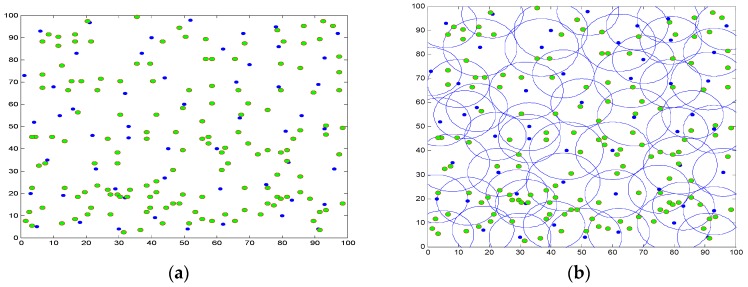
The distribution and coverage of active nodes after sleep scheduling. (**a**) Dormant nodes’ deployment; (**b**) Coverage area.

With the operation of the network, the node scheduling scheme determines redundant nodes gradually. Figure 7a,b shows the distribution and coverage of active nodes after sleep scheduling. The sign “o” in Figure 7 represents the dormant node, and the number of dormant nodes arrives 157, which is about 78.5% of the total number. It can be seen that a large number of redundant nodes are scheduling into sleep state, and active nodes can still ensure the full coverage of the monitoring area, which can greatly save network energy overhead.

**Figure 8:**
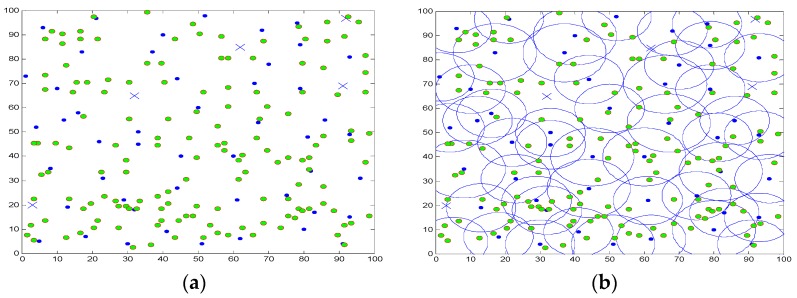
The distribution and coverage while some active nodes failed. (**a**) Some nodes being depleted; (**b**) Coverage hole.

With the gradual depletion of the energy of some nodes, Figure 8 shows the situation while some active nodes failed in the network, where “x” indicates the failed node. During the current round, there are five active nodes being labeled failed state due to energy depletion, and the network can no longer be able to achieve full coverage of the region.

**Figure 9:**
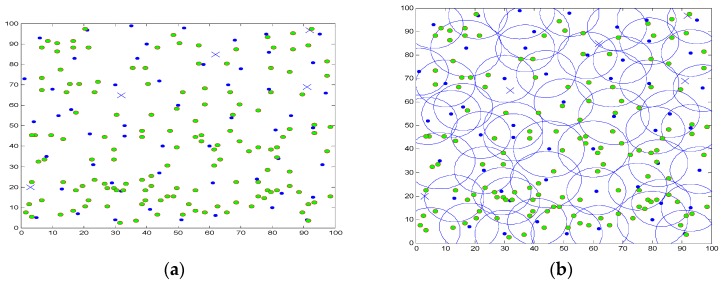
The distribution and coverage after some hibernated node being awaken. (**a**) Some hibernated node being awaken; (**b**) Coverage area.

Figure 9 shows that the relevant dormant nodes will be awaken to repair coverage holes. It can be seen that there are four dormant nodes being awakened, and the network retains high target area coverage, it can be observed that the CPCSS can effectively control the working status of nodes according to the network variation, so as to achieve the goal of saving energy consumption. In addition, the adjacent nodes in sleep state can be waken up timely to maintain high coverage of the system as soon as the appearance of some nodes failed due to energy depletion.

**Figure 10:**
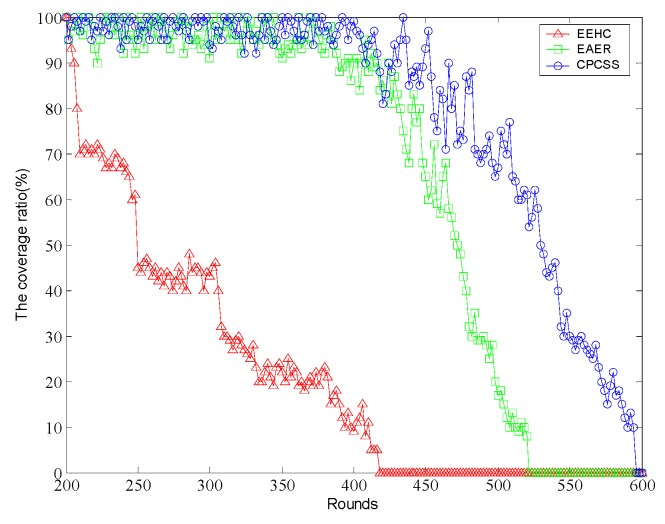
Comparison of the coverage ratio over rounds.

Figure 10 shows the coverage of the algorithm over rounds. For EEHC, if not carrying out any scheduling scheme, that is to say, all nodes are active and it can get high network coverage at the beginning of system running. However, with the increase of the number of rounds, the coverage will demonstrate a sharp drop. 

The coverage rate of the EAER algorithm also decreases gradually over rounds, and the coverage rate of the CPCSS algorithm is much slower than others. At 518 rounds of the network, the EAER coverage is close to zero, and at this time the coverage ratio in CPCSS is still 61.2%. Until 591 rounds, the coverage rate of the CPCSS algorithm will drop to zero rather slowly. It is mainly beneficial of that each node can dynamically adjust its own state according to the residual energy and the coverage of the neighbor nodes in CPCSS. In EAER, the periodic asynchronous mode is repeated in the state estimation, which has a great effect on the energy consumption and reduces the energy efficiency.

**Figure 11:**
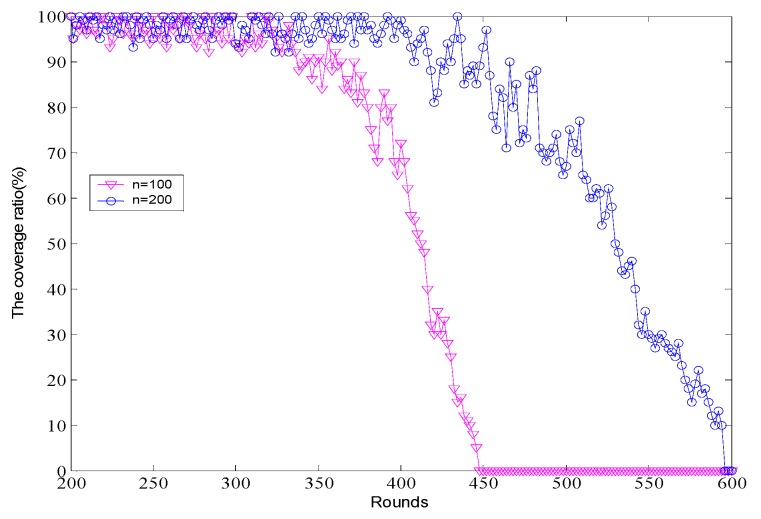
Comparison of the coverage ratio over rounds.

The performances of the proposed algorithm with 100 and 200 nodes uniformly deployed on a network field are shown in Figure 11. In the simulation scenario, the sensing range of the node is within the limits of 8 m to 12 m while the corresponding communication range of each node is about 16–24 m, and the other parameters are consistent with the simulation parameters in the previous experiments. As expected, sleep scheduling algorithms provide a remarkable gain and maintain a high coverage rate long-term with the network running. No matter how the network density changes, the high coverage of the target area can be guaranteed during the most of the lifetime of network.

**Figure 12:**
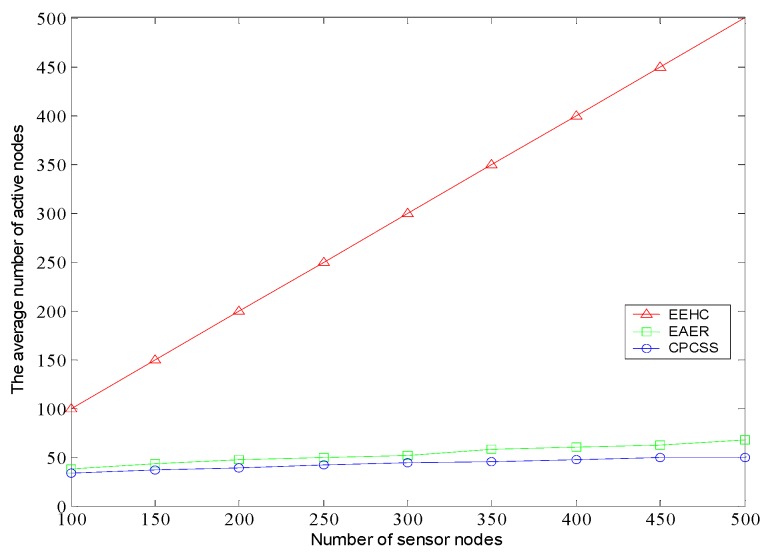
The average number of active nodes versus number of sensor nodes.

Figure 12 shows that the number of active nodes varies with the increase of the initial deployment node. As it can be seen, the number of active nodes increases with a different extent in case of different density of nodes deployed from 100 to 500. Theoretically, the number of active nodes required for sensing the target area should be approximately constant. 

The main reason for this phenomenon is the boundary effect of the target region, in which the nodes at the edge of the area usually have less chance to be hibernated nodes owing to hard to be replaced. When the number of initial nodes is 100, active nodes in EAER and CPCSS are 38 and 33 respectively, the latter can reduce about 13.16% of active node’s deployment in the network. While the total number of nodes is 300, EAER need to deploy 52 active nodes, is much higher than 44 in CPCSS. In EEHC, all nodes are active which will result in energy depletion in advance.

**Figure 13:**
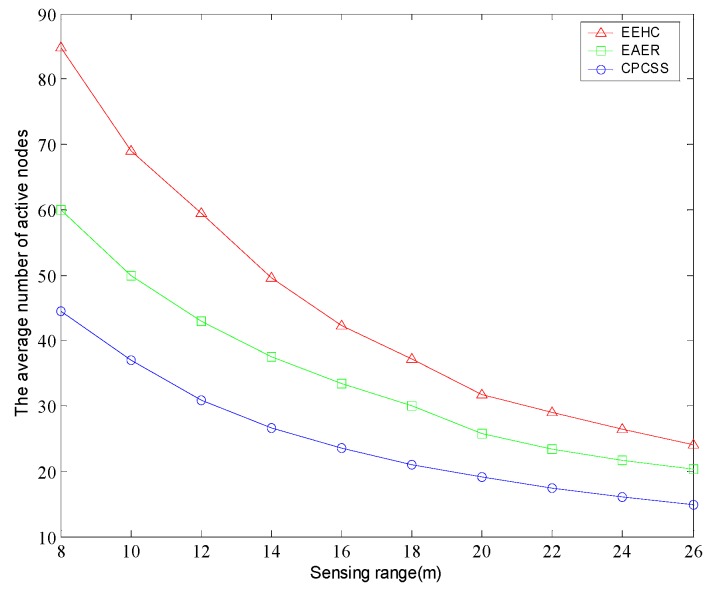
The average number of active nodes versus sensing range.

Figure 13 shows that the average number of active nodes in the network varies with the sensing range. With the increase of the node sensing range, the number of active nodes of the algorithms is gradually reduced in the premise of ensuring the network coverage. This is because the greater the sensing range of the node, less active nodes is required to achieve the same coverage effect; namely, fewer nodes can retain the network coverage. Simulation results indicate that the number of active nodes in CPCSS is less than EAER in different sensing range model, and the advantage is more obvious especially the sensing range is small.

**Figure 14:**
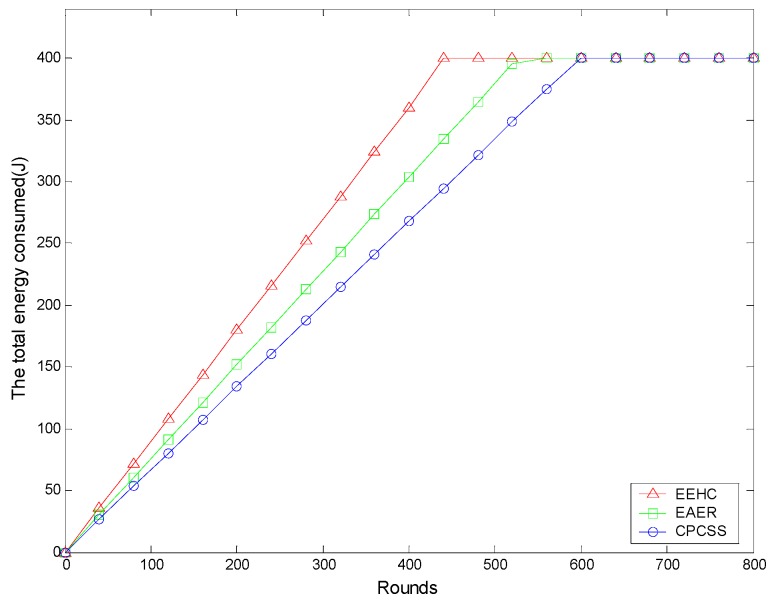
The total energy consumed over rounds.

To investigate the sensor’s performance in terms of energy consumption, we compare the energy consumption of the nodes in CPCSS, EAER, and EEHC. Figure 14 shows the variation of total energy consumption of the network over the number of rounds. Since the initial energy of each node is 2 J, there are 200 nodes being deployed in the network and the total energy is 400 J. As can be seen from the results, the energy consumption of CPCSS is much slower than the EEHC and EAER.

**Figure 15:**
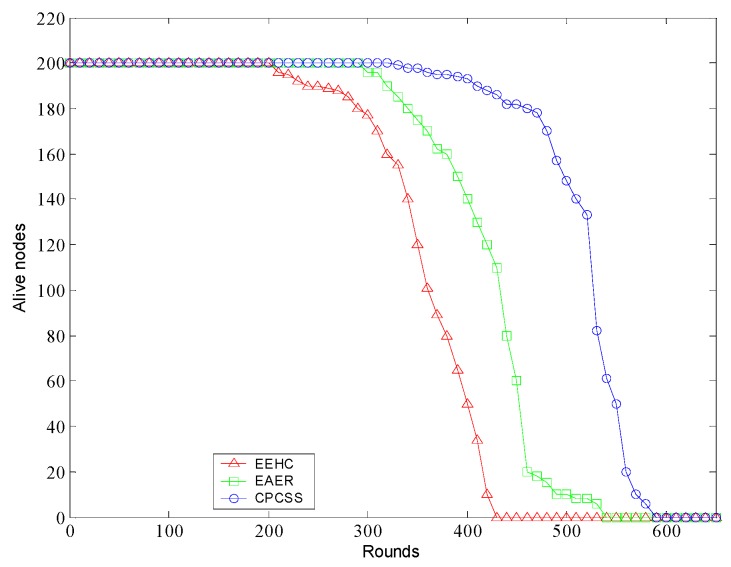
The alive nodes over rounds.

Figure 15 shows the number of alive nodes over rounds. It can be seen that in contrast to the EAER and EEHC protocols, the number of alive nodes in CPCSS decreases more smoothly. This is because the reduction of working sensors in CPCSS can conserve energy and prolong their operation lifetimes.

**Figure 16:**
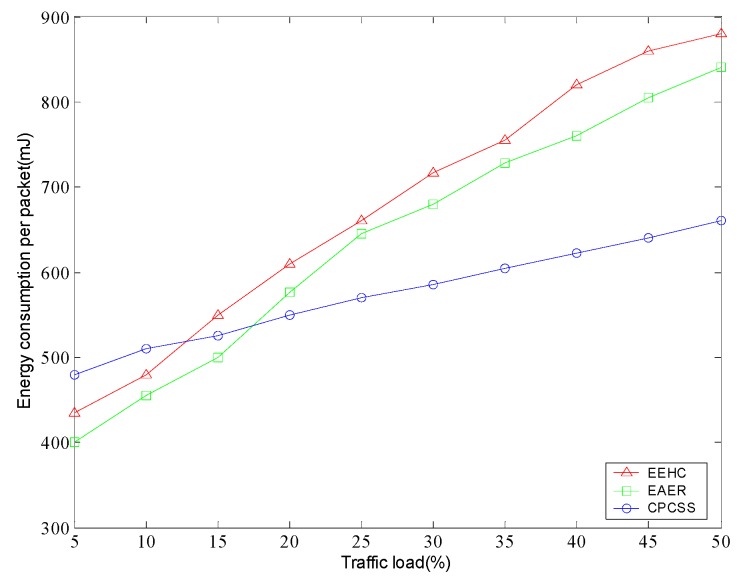
Energy consumption per packet in different traffic load.

Subsequently, we examine the performance of energy efficiency of the whole network. Figure 16 depicts the comparison of energy consumption per packet in different protocols. From the simulation results, we can find that the energy consumption per packet of all methods monotonously increases with the traffic load. Also, it can be observed that CPCSS does not always show the better performance than the other protocols. As the traffic load is relatively low, CPCSS consumes more energy for each packet due to excessive overhead for node classification and redundancy calculation. Along with the traffic load of network increases, this type of impact turns out to be negligible in contrast of communication overhead and CPCSS can demonstrate high energy efficiency.

## 6. Conclusions

This paper addresses the problem of energy efficiency enhancement and coverage quality assurance in high-density ESNs, and a coverage-preserving control scheduling scheme based on cloud model in emerging sensor networks is proposed. Our solution exploits the correlations of the data monitored by the adjacent sensor nodes to classify the nodes into different categories, and then generates the redundant nodes set for sleep scheduling. Experimental results demonstrate that an effective sleep-wake cycle scheduling algorithm can improve the efficiency of the whole system and optimize the connectivity and coverage performance. In the future, we will study the use of sparse over-complete representation for data similarity in ESNs, and the work will address enhancing the performance of the proposed technique in settings with sparse deployments or mobile sensor nodes.
